# Cell Wall Proteome Profiling of a *Candida albicans* Fluconazole-Resistant Strain from a Lebanese Hospital Patient Using Tandem Mass Spectrometry—A Pilot Study

**DOI:** 10.3390/microorganisms9061161

**Published:** 2021-05-28

**Authors:** Andy Awad, Pamela El Khoury, Geovanni Geukgeuzian, Roy A. Khalaf

**Affiliations:** Department of Natural Sciences, Lebanese American University, Byblos P.O. Box 36, Lebanon; andy.awad@lau.edu (A.A.); pamela.elkhoury@lau.edu.lb (P.E.K.); Geovanni.Geukgeuzian@lau.edu (G.G.)

**Keywords:** *Candida albicans*, mass spectrometry, fluconazole, azole resistance, cell wall

## Abstract

*Candida albicans* is an opportunistic pathogenic fungus responsible for high mortality rates in immunocompromised individuals. Azole drugs such as fluconazole are the first line of therapy in fungal infection treatment. However, resistance to azole treatment is on the rise. Here, we employ a tandem mass spectrometry approach coupled with a bioinformatics approach to identify cell wall proteins present in a fluconazole-resistant hospital isolate upon drug exposure. The isolate was previously shown to have an increase in cell membrane ergosterol and cell wall chitin, alongside an increase in adhesion, but slightly attenuated in virulence. We identified 50 cell wall proteins involved in ergosterol biosynthesis such as Erg11, and Erg6, efflux pumps such as Mdr1 and Cdr1, adhesion proteins such as Als1, and Pga60, chitin deposition such as Cht4, and Crh11, and virulence related genes including Sap5 and Lip9. Candidial proteins identified in this study go a long way in explaining the observed phenotypes. Our pilot study opens the way for a future large-scale analysis to identify novel proteins involved in drug-resistance mechanisms.

## 1. Introduction

Although part of the normal microbiome in humans, *Candida albicans* (*C. albicans*) can switch from a harmless commensal fungus to a human pathogen causing infections that range from superficial oral or vaginal candidiasis to life-threatening systemic infections [1]. Risk factors of *Candida* infections include diabetes, pregnancy, neutropenia, and immunocompromization following intravenous catheterization, chemotherapy, transplantation, invasive procedures, prolonged use of broad-spectrum antibiotics, or AIDS [2,3]. *C. albicans* is classified as the major etiological agent of invasive fungal infections [4]. It is considered a critical public health concern as it imposes economic and medical burdens such as high costs of care, high mortality rate, and extended hospitalization duration [5].

Typically, treatment of *C. albicans* infections is limited to five major classes of antifungal drugs: azoles, polyenes, fluoropyrimidines, allylamines, and echinocandins [6]. Azole antifungals are usually the most preferred drugs for treating candidiasis as they have limited toxicity, are relatively cheap, and are administered orally [7]. The azole drug fluconazole is the most commonly used antifungal [8]. As with other azoles, fluconazole suppresses lanosterol 14-α-demethylase [9]. This enzyme is encoded by *ERG11* and normally functions in catalyzing lanosterol to C14-demethyl-lanosterol and consequently synthesizing ergosterol which is a main component of the cell membrane [10]. By inhibiting the demethylation of lanosterol, fluconazole inhibits ergosterol biosynthesis and causes the accumulation of methylated sterols in the cellular membrane, thereby disrupting the cell membrane and arresting fungal growth [11,12]. Since fluconazole is fungistatic and not fungicidal, treatment permitted the development of acquired resistance among some *C. albicans* strains in the presence of fluconazole [13]. The prolonged and widespread use of fluconazole, and azoles in general, increases the risk of multidrug resistance that is a critical concern in antifungal therapy [14].

The onset of resistance to antifungals is clinically important and is controlled by several mechanisms. For instance, point mutations in *ERG11* result in the reduced ability of the enzyme to bind fluconazole [15]. Another mechanism is through the overexpression of *ERG11* leading to increased ergosterol biosynthesis [16]. Furthermore, gain-of-function gene mutations in some transcription factors lead to constitutive up regulation of numerous target genes and increased fluconazole resistance. For example, mutations in Mrr1 and Tac1 constitutively overexpress the efflux pumps *MDR1* and *CDR1*/*CDR2*, respectively, which considerably enhance fluconazole resistance in *C. albicans* through decreasing intracellular drug accumulation [17]. Additional mechanisms include alterations in enzymes involved in the ergosterol biosynthetic pathway, changes in plasma membrane components and cell wall proteins (CWPs) types, and lowered uptake of fluconazole, in addition to other mechanisms yet to be elucidated [18]. 

We have previously obtained drug-resistant and sensitive *C. albicans* isolates from local Lebanese medical centers. One of these strains was found to be resistant to all azoles tested [19]. We recently subjected the strain to multiple phenotypic assays to characterize it. The strain exhibited reduced growth, a 26% increase in chitin content, 70% increase in membrane ergosterol content, and a 44% decrease in biofilm formation when compared to the reference *C. albicans* strain SC5314. It was also found to be overly adherent, but with a decreased resistance to cell wall-disrupting agents. *ERG11* sequencing indicated frame-shift mutations and a disseminated virulence assay revealed that the strain was slightly attenuated in virulence compared to the reference strains [20]. Since fluconazole resistance is the result of the up-regulation of efflux pumps that are localized to the cell surface and differential expression of many CWPs, we applied a proteomic approach to our fluconazole-resistant *C. albicans* strain to identify CWPs expressed upon exposure to fluconazole. Such CWP might explain the mechanisms behind the above-mentioned phenotypes. This pilot study will be expanded in the future to include fluconazole-sensitive strains and compare their proteomic profiles to fluconazole resistant ones with the aim of detecting differentially expressed proteins involved in resistance acquisition mechanisms. 

## 2. Materials and Methods

Strains used: One fluconazole-resistant *C. albicans* strain (MIC > 256 μg/mL) was utilized in this study [19]. 

Culture Conditions: RPMI 1640 media (with L-glutamine and no bicarbonate) with MOPS and glucose (AB Biodisks) was prepared according to the manufacturer’s instructions. The strain was grown at 28°C on potato dextrose agar plates (Oxoid) supplemented with histidine and uridine. *C. albicans* colonies were suspended in 0.154 M sterile saline solution up to a turbidity of 0.5 McFarlans and were spread over RPMI 1640 plates with a sterile cotton swab. A fluconazole E-test strip (AB Biodisk, Solna, Sweden) was then placed at the center of each RPMI 1640 plate and the plates were incubated for 48 h at 28 °C. 

Cell harvesting: Fluconazole-resistant cells were harvested from the edges of the fluconazole strip outwards at high concentrations, ensuring that the harvested cells were properly exposed to the antifungal drug.

Cell wall isolation and protein extraction: Eight independent cell wall extractions were performed as described previously [21]. A minimum weight of 50 mg of cultured cells was collected and the cells were washed with water at least three times, centrifuged at 4000 rpm for 5 min, and then re-suspended in 5 mL Tris (5 mM, pH = 7.8). They were then divided into 1 mL Eppendorf tubes and centrifuged for 3 min at 12,000 rpm. Pellets were resuspended in 750 μL Tris (5 mM, pH = 7.8). Protease inhibitor cocktail (6 μL, Abcam ab65621) and cold glass beads were added (beads-to-pellets ratio of 1:1). Thirty vortexing cycles on a bead beater were performed for breakage as follows: 30 s on vortex then 30 s on ice. Orange color resulting from the reaction between the acidic cytosol and the protease inhibitor signifies breakage and the efficiency of the breakage was monitored using microscopy. The solution on top of the beads was transferred to new pre-weighed tubes. The beads were washed multiple times using cold NaCl (1 mM) and poured again over the respective pre-weighed tubes to collect as much cell wall material as possible without transferring any beads during the procedure. Samples were centrifuged at 3000 rpm for 5 min. The supernatant containing intracellular proteins was poured off while the pellet was washed in NaCl (40 mL, 1 mM) at least three times. SDS extraction buffer (50 mM Tris, 2% SDS, 100 mM Na-EDTA, 150 mM NaCl, pH 7.8) with β-mercaptoethanol (β-ME) (8 μL per 1 mL SDS extraction buffer) was added (0.5 mL buffer per 100 mg wet weight walls). Tubes were boiled for 10 min then cooled down to room temperature. They were centrifuged for 5 min at 3000 rpm and the supernatant was collected and labeled SDS-extracted protein fraction to be later subjected to tryptic digestion. SDS extraction buffer and β-ME were added as before to resuspend the pellet. The tubes were boiled and cooled down to room temperature three more times then centrifuged for 5 min at 3000 rpm and the resulting pellet was suspended and washed with water to remove excess SDS. The final pellet was frozen in liquid nitrogen and freeze-dried. Lyophilized cell walls were stored at 20 °C until use.

Extraction of alkali labile CWPs: Cell wall pellets were incubated overnight with NaOH (30 mM) at 4 °C and then neutralized with aqueous acetic acid (30 mM) [22]. They were centrifuged; supernatants were collected and subjected to tryptic digestion.

Extraction of CWPs using Glucanase: Cell wall pellets were treated with 1 mg of B-(1-3)-D-Glucanase from *Helix pomatia* (Sigma-Aldrich 67138, specific activity ≥ 0.2 U/mg) in sodium acetate buffer (1 mL, 150 mM, pH = 5) per 10^8^ cell equivalents and were incubated overnight at 37 °C [23]. Cell numbers were estimated using spectrophotometric analysis at 600 nm. Supernatants were collected and subjected to tryptic digestion.

Tryptic digestion: CWP fractions were incubated in a reducing buffer (10 mM DTT, 100 mM NH_4_HCO_3_) at 55 °C for an hour then spun and pellets were resuspended in an alkylating buffer (65 mM iodoacetamide, 100 mM NH_4_HCO_3_). They were incubated for 45 min at room temperature in the dark. A quenching solution (55 mM DTT, 100 mM NH_4_HCO_3_) was added to the samples and kept for five minutes at room temperature followed by 5× washing with ammonium bicarbonate buffer (50 mM). The pellets were re-suspended in a solution containing ammonium bicarbonate (50 mM) and trypsin (1 μg/μL) and left at 37 °C for 16 h. Finally, they were spun, and the supernatants were collected. TFA (0.1% *v/v*) was added to prepare samples for subsequent ZipTip clean-up [24].

ZipTip clean-up: ZipTip C18 clean-up tips (Millipore^®^ Ziptips, Sigma-Aldrich, volume 10 μL, 0.6 μL C18 resin) were wetted in acetonitrile solution. They were equilibrated in HPLC water solution containing 0.1% TFA. Sample binding was achieved by full pressing at least 10 times in the sample tubes. The tips were then washed in HPLC water solution containing 0.1% TFA. The samples were eluted using 10 μL of elution buffer (0.1% TFA (*v/v*) in HPLC water/acetonitrile (1:1)).

Tandem mass spectrometry: Concentrated and cleaned-up peptides were spotted on a stainless-steel target plate (Opti-TOF TM 384 Well Insert, 128 × 81 mm RevA, Applied Biosystems). They were overlaid with α-cyano-4-hydroxy-cinnamic acid matrix solution (10 mg CHCA matrix in 50% acetonitrile with 0.1% TFA) and air-dried. MALDI-TOF-TOF MS spectra were acquired using the 4800 MALDI-TOF-TOF analyzer, operated by the 4000 Series Explorer software (version 3.7). The instrument was externally calibrated using TOF/TOF Calibration Mixture (Mass Standards Kit for Calibration of AB SCIEX TOF/TOF Instruments). MS reflector positive mode was used for acquisition at a laser intensity of 2500. The mass range was set at 499 to 2500 Da and the focus mass was set at 1500 Da. The reflector positive default was used as a processing method with a minimum signal-to-noise ratio of 5. The resulting mass lists were manually scanned for known contaminant mass peaks (keratin, matrix, and trypsin autolysis) and an exclusion list was created to be applied for all performed MS/MS data acquisitions. For the interpretation method, MS/MS 1 kV positive was utilized as an MS/MS acquisition method specifying a fixed laser intensity of 3500 and a precursor mass of 1570.677 Da. Metastable suppressor and CID were turned on. MS/MS positive default was utilized as an MS/MS processing method in which a signal-to-noise threshold of 5 for monoisotopic peaks was chosen.

Protein identification: MS/MS Ion Search was performed first using the common Repository of Adventitious Proteins, cRAP, database to eliminate possible contaminants whose peaks were not inserted in the exclusion list. Then, a custom *C. albicans* CWP database on the MASCOT server was used in order to identify the proteins within the samples as described previously [24]. The database includes sequences of all curated *C. albicans* proteins available in the Swissprot database with gene ontology related to cell wall, plasma membrane, and transmembrane localization tags. As such, the database contains a total of 218 sequences to be searched. In the MASCOT Server’s search parameters page, peptide and fragment tolerance values were set at 2 Da each, “Carbamidomethyl C” and “Oxidation M” were chosen as fixed modification and variable modification, respectively, and up to two missed cleavages were allowed for trypsin. Moreover, a peptide charge of 1+ was allocated and MALDI-TOF-TOF was selected in the instrument type option. In the peptide summary report obtained by MASCOT, individual ions scores greater than 15 indicated identity or extensive homology and proteins were considered successful hits if they had a minimum sequence coverage of 2% or if their peptide sequence was at least 12 amino acids long as these are sufficient for unmistakable protein identification [25]. Peptide sequences provided by MASCOT but unassigned to any protein were identified via BLAST search in the *Candida* Genome database (www.candidagenome.org (accessed on 10 January 2021) in which the chosen target genome and target sequence dataset were “*Candida albicans* SC5314 Assembly 22” and “Proteins–translation of coding sequence (PROTEIN)”, respectively, and no gapped alignments were permitted. The cutoff E-value was set at <0.05 for both MASCOT and BLAST searches. The STRING database (version 11) was used to find a protein–protein interaction prediction map of detected proteins [26]. The confidence score and false-discovery rate (FDR) stringency were set at 0.400 and 5%, respectively, and the chosen organism was *Candida albicans*. The STRING database was also used to highlight proteins with the following biological processes: drug metabolic process (GO: 0017144), cellular response to drug (GO: 0035690), response to fungicide (GO: 0060992), and/or ergosterol biosynthetic pathway (GO: 0006696).

## 3. Results

In total, 50 proteins were successfully identified in our fluconazole-resistant *C. albicans* strain: 44 proteins were identified using MASCOT search while the resultant unmatched sequences allowed the identification of additional six proteins using BLAST search.

### 3.1. Essential Proteins and Proteins Involved in Growth

Out of the 50 detected proteins, six were found to be essential proteins for the normal growth, survival, and pathogenicity of *C. albicans*: Ipp1, Ape2, Pan1, Eft2, Eno1, and Hsp90. Additional proteins involved in cytokinesis, budding, glycolysis or other processes required for growth were also detected: Mts1, Gpm1, Bud4, and Eng1. More details concerning the essential proteins and proteins functioning in growth are shown in Table 1.

### 3.2. Proteins Involved in Adhesion

The major adhesin, Als1, and other proteins with roles in the adhesion of *C. albicans* to biotic and abiotic surfaces were identified in our study. Those proteins explain the adherent phenotype of the studied strain and are presented in Table 2.

### 3.3. Proteins Involved in Cell Wall Integrity and Chitin Content

Chitin binding and remodeling proteins such as Cdc11, Utr2, and Cht4 were detected. Furthermore, proteins involved in plasma membrane destabilization (End3) and cell wall organization (Pga41, Phr1, Phr2, Crh11, Utr2) were identified. Identified proteins with roles in cell wall integrity and chitin content are presented in Table 3.

### 3.4. Pumps and Transporters

We were able to detect two important efflux pumps from two different classes: Mdr1 and Cdr1. Those proteins play significant roles in fluconazole resistance. Additionally, Mlt1, a multiple drug resistance-associated transporter was detected. All three proteins were detected using MASCOT and are presented in Table 4.

### 3.5. Proteins Involved in Ergosterol Biosynthesis and Iron Acquisition

Erg11, Erg6, and Bna4 are three proteins detected in our fluconazole-resistant strain that has increased ergosterol content in its membrane. Moreover, proteins involved in iron acquisition (Pga10, which is also known as Rbt8, and Csa1) were detected. These proteins were identified using MASCOT and their details are provided in Table 5.

### 3.6. Proteins Involved in Virulence

Results showed that only one secreted lipase (Lip9) and one secreted aspartyl protease (Sap5) were detected in the studied fluconazole-resistant *C. albicans* strain. Yet, other proteins contributing to nutrient acquisition (Stp2 and Stp3), and other virulence attributes were also detected and presented in Table 6.

### 3.7. Proteins Involved in Other Functions

Some of the identified proteins are not yet characterized or their function has not been identified yet (Pga30, Pns1, C1_13530W_Ap). Some atypical proteins were also identified, especially those with known functions in the mitochondria (Pam17, Ssc1, Mic60, and Aim36) and Tsa1b. It is interesting to note the presence of Pil1, which is an echinocandin-binding protein and a component of the eisosome. Table 7 lists the proteins involved in different functions than the ones listed above in addition to uncharacterized proteins.

### 3.8. Protein-Protein Interactions

**Figure 1:**
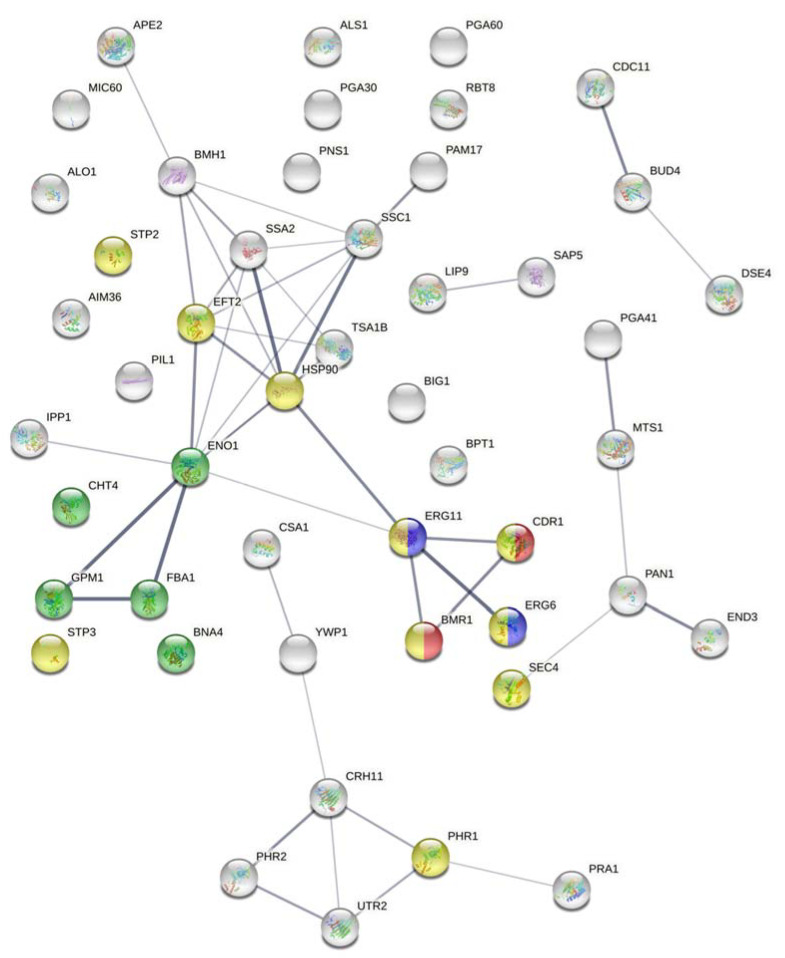
Prediction map of the protein–protein interactions among the identified cell wall proteins in the fluconazole-resistant *C. albicans* strain. The map was constructed using STRING database (version 11.0) with a minimum confidence score of 0.4 where line thickness reflects the strength of data support. Joint proteins function in interconnected pathways. Colored nodes refer to proteins with biological process related to drug metabolic process (green), cellular response to drug (yellow), response to fungicide (red), and/or ergosterol biosynthetic pathway (blue). Filled nodes refer to proteins with known or predicted structures whereas empty nodes refer to proteins with unknown structures.

A prediction map of the protein–protein interactions among the identified proteins in the fluconazole-resistant strain was obtained. Identified proteins are involved in interconnected pathways such as steroid biosynthesis, biosynthesis of antibiotics, glycolysis or gluconeogenesis, and metabolic pathways. Most importantly, the map showed which proteins are involved in biological processes related to antifungal therapy: the drug metabolic process (Eno1, Cht4, Gpm1, Fba1, and Bna4), cellular response to drug (Stp2, Stp3, Eft2, Hsp90, Phr1, Sec4, Erg6, Erg11, Mdr1, and Cdr1), response to fungicide (Cdr1 and Mdr1), and ergosterol biosynthetic pathway (Erg6 and Erg11). The prediction map is presented in Figure 1.

## 4. Discussion

The cell wall in a fungus is antigenic as it is the first structure that contacts the host. It includes and incorporates proteins involved in adhesion, biofilm formation, virulence and drug resistance [24]. This study aimed to determine the cell wall proteomic profile of a previously characterized fluconazole-resistant *C. albicans* strain exposed to fluconazole in order to identify proteins involved in azole resistance and pathogenicity-related phenotypes. We have previously successfully applied such a technique in identifying proteins differentially expressed in cell wall mutants [21,24]. In this context, we are applying it to determine changes in cell wall protein composition in response to drug exposure. The previous characterization of our resistant strain found a 44% reduction in biofilm formation, a 26% increase in chitin content, and a 70% increase in ergosterol content compared to the reference strain [20]. The strain was also hyper-adherent, and slightly less virulent. Since the cell wall is the initial point of contact between the fungus and its host, its composition is dynamic, and CWP expression varies depending on specific environmental stresses and cues [27]. We hypothesized that a fluconazole-resistant strain under fluconazole exposure results in the expression of proteins and in changes at the level of the cell wall structure that explain our previously observed phenotypes. CWP extraction following fluconazole exposure coupled with tandem mass spectrometry and database mining revealed such proteins.

In total, 50 proteins associated with the cell surface were identified in this study. Interestingly, we were able to identify some atypical proteins otherwise referred to as moonlighting proteins since they are of cytoplasmic origin yet are also detected at the cell surface with a different yet important role in fungal virulence such as Fba1, Gpm1, Ipp1, and Eno1 [28]. Gpm1 has been reported to be an important virulence factor in *C. albicans* as it is involved in degradation of the extracellular matrix and host immune evasion and was found to be induced by fluconazole [29,30]. Ipp1 and Eno1 are also essential proteins. The latter has a role in the growth, morphogenesis, cell division and osmotic protection of the fungus [31]. Another atypical protein is Tsa1B, a peroxidase that plays a major role in resisting heat shock and oxidative stress [32]. Additionally, proteins with known functions in the mitochondria (Pam17, Ssc1, Mic60, and Aim36) were detected; they could either be artifacts resulting from contamination during cell wall isolation or atypical proteins with unclear functions in the cell wall. The essential protein, Eft2, is antigenic and was assigned as an atypical protein in a previous study [33]. It is induced upon exposure to stresses and is the target of sordarin antifungals [34]. Additional essential proteins were identified such as Ape2, Pan1, and Hsp90. Proteins involved in cytokinesis, budding, glycolysis or other processes required for growth were also detected: Eng1, Bud4, and Mts1. The identified heat shock proteins (Hsp70 and Hsp90) play roles in multiple cellular pathways and consequently confer drug resistance [35]. These proteins identified in the fluconazole-resistant strain explain its ability to survive in the presence of fluconazole. Yet, the low number of detected essential proteins could explain its growth at a lower rate.

Proteins promoting adhesion were detected in our fluconazole-resistant strain such as Big1, Pga60, and Als1. One of the most important adhesins in *C. albicans*, Als1, is crucial for fungal adherence and aggregation and was recently linked to fluconazole resistance [36]. In addition to their roles in cell wall assembly, the glycosidases Utr2 and Crh11 were found to be involved in the adhesion of *C. albicans* to host cells [37,38]. As such, these proteins explain the adherent phenotype previously observed in the studied strain [20]. However, Ywp1, which is an anti-adhesive protein only expressed in the yeast form and thought to function in yeast cell dispersal to colonize new sites, was also detected [39]. Yeast cells lacking this protein were found to form thicker biofilms than their counterparts [40]. This could explain the previously observed 44% decreased biofilm ability of our fluconazole-resistant strain. Another protein localized to yeast cells and not to hyphae is Bmh1. Knowing that fluconazole hinders hyphal formation, the observed phenotype of reduced biofilm formation ability is not surprising especially since biofilm formation requires both yeast and hyphal forms [41]. The studied fluconazole-resistant strain could still form biofilms due to the presence of Csa1and Pga10, which play a crucial role in biofilm formation, development and maintenance [42].

Plasma membrane fluidity is increased following fluconazole treatment which could affect the integrity of the cell wall and lead to increased chitin content as a compensation; our strain was found to exhibit a disrupted cell wall following SDS treatment and a 26% increase in chitin content [20]. The identified glycosidases (Crh11 and Utr2) and pH-responsive proteins (Phr1 and Phr2) are induced by fluconazole exposure and they are involved in cell wall cross-linking and cell wall integrity [38,43]. Furthermore, the identified proteins involved in endocytosis (Pil1 and End3) could explain the increased fluidity caused by fluconazole exposure [43]. In addition, Pil1 is involved in echinocandin binding. Its presence here is interesting since our strain is sensitive to the echinocandin caspofungin, suggesting a possible role for Pil1 in azole resistance. The septin Cdc11 is involved in chitin deposition and the chitinase Cht4 functions in chitin remodeling [44,45], thus explaining the increased chitin content in our strain.

The fungal cell wall usually incorporates many virulence factors including lipases, secreted aspartyl proteases, and superoxide dismutases that enable the fungus to acquire nutrients from its extracellular environment, invade the host, and escape host defenses [1]. In our strain, we only detected Lip9 and Sap5. In addition, the transcriptional regulators Stp2 and Stp3 that activate genes encoding lipases were detected and are involved in virulence [46]. The low number of virulence genes detected and the absence of other key virulence proteins could be the reason for the reduced virulence ability of the strain.

We previously demonstrated that the resistant-fluconazole *C. albicans* strain had a frameshift in *ERG11* generating a protein that cannot bind to fluconazole and resulting in resistance. Erg11 overexpression might be another mechanism contributing to fluconazole resistance in our strain, especially since Erg11 was detected with relatively high coverage. In addition to Erg11, Erg6 and Bna4, which play roles in ergosterol biosynthesis, were also detected. This is in agreement with studies showing an increased abundance of Erg6 in fluconazole-resistant *C. albicans* strains upon exposure to fluconazole [47]. Since these proteins function in ergosterol biosynthesis, their presence explains the observed 70% increase in ergosterol content. It has been suggested that the need for iron increases during azole stress, especially given that iron is required by multiple enzymes acting in the ergosterol biosynthetic pathway [43]. Csa1 and Rbt8 are two identified proteins functioning in heme-iron utilization [42]. Another mechanism for acquiring fluconazole resistance is through incorporating efflux pumps [17]. We were able to detect two major efflux pumps (Mdr1 and Cdr1) in our studied fluconazole-resistant strain. Mdr1 is a major facilitator superfamily (MFS) efflux pump while Cdr1 is a pleiotropic ATP-binding cassette (ABC) efflux transporter; they transport azoles out of the cell, thus reducing drug accumulation intracellularly and permitting resistance [48,49].

Moreover, we found that some of the identified proteins are involved in interrelated pathways such as the biosynthesis of steroids and antibiotics, glycolysis, and metabolic pathways. We were also able to identify some proteins involved in the drug metabolic process (Eno1, Cht4, Gpm1, Fba1, and Bna4), cellular response to drug (Stp2, Stp3, Eft2, Hsp90, Phr1, Sec4, Erg6, Erg11, Mdr1, and Cdr1), response to fungicide (Cdr1 and Mdr1), and ergosterol biosynthetic pathway (Erg6 and Erg11).

For a comparison between fluconazole-resistant and sensitive *C. albicans* strains, we present the preliminary MASCOT results of the same cell wall extraction method applied on one sensitive strain. A total of 20 proteins were identified, four of which were common with the studied fluconazole-resistant strain: Hsp90, Phr1, Sec4, and Pam17. Since both strains are grown in the presence of fluconazole and knowing that Hsp90 and Phr1 are part of the cellular response to the drug, the detection of these two proteins in both strains is not surprising. Some of the proteins solely detected in the sensitive strain were constituents of the mitochondria and endoplasmic reticulum, such as Pam18 and Irc22-1, respectively. Rps1 is a constituent of the ribosome and was shown to elicit a host antibody response during infection [50]. Additionally, we detected Kex2 in the fluconazole-sensitive strain, which is a subtilisin-like protease that processes the aspartyl proteinase Sap2 and is needed for virulence and hyphal growth of *C. albicans* [51]. Several important mannosyltransferases were detected solely in the fluconazole-sensitive strain, such as Alg1, Rhd1, Mnn1, Mnn12, and Mnn13. The proteins Alg1 and Rhd1 are beta-mannosyltransferases needed for growth and cell wall biosynthesis [52,53]. Mnn1, Mnn12, and Mnn13 are alpha-mannosyltransferases involved in hyphal development, biofilm formation, and virulence [54]. Manipulation of the mannan layer and composition increases cell-to-cell adhesion and provides protection from environmental stresses such as antifungals [55]. These proteins were present in the fluconazole-sensitive strain and therefore explain its increased virulence, colonization, and biofilm forming ability when compared to the fluconazole-resistant strain. Likewise, their lack of detection in the fluconazole-resistant strain could explain its attenuated virulence and defect in biofilm formation. Another important protein detected exclusively in the sensitive strain is Pga14. This protein is a hydrophilin, induced during cell wall regeneration and it is critical for overcoming the stress of desiccation-rehydration process [56]. The remaining proteins exclusively identified in the fluconazole-sensitive strain were: Rcf1, Cef3, Pga43, Lcl3, Pfa5, and Tef1. No efflux pumps or key proteins in ergosterol biosynthesis were detected in this fluconazole-sensitive strain, thus implying the role of these proteins in conferring resistance to fluconazole.

In summary, this pilot study successfully detected CWPs expressed in a fluconazole-resistant strain upon exposure to fluconazole. Most of the cell wall proteins that were expressed and identified belong to pathways involved in resistance and pathogenicity in accordance with our previously observed phenotypes, a testament to the dynamic nature of cell wall protein expression and deposition in response to fluconazole stress. Some of these identified proteins can be targets of novel therapeutic strategies. Future work will expand on this study and investigate differential protein expression between resistant and sensitive strains to better elucidate the role that azoles play in modulating specific cell wall protein expression. Similar studies have previously been carried out in our lab to compare *C. albicans* mutant with wild-type strains under filamentous and non-filamentous conditions [21,24,57,58,59].

## Figures and Tables

**Table 1:** Essential proteins and other proteins involved in growth were identified in the fluconazole-resistant *C. albicans* strain. Proteins identified using MASCOT are represented with sequence coverage while proteins identified using BLAST are represented with E-value.

Protein Accession	Gene Name	Protein Description	MASCOT Score	Sequence Coverage (%)	Missed Cleavage	Peptide Sequence	E-Value
P83777	*IPP1*	Inorganic pyrophosphatase	16	5.6	0	VLGVMALLDEGETDWK	-
Q59KZ1	*APE2*	Aminopeptidase	40	3.5	0	ASYQEDGK	-
					0	INGDQSGIYR	-
					1	YMATTQMEPTDCRR	-
Q5AHB1	*PAN1*	Actin cytoskeleton-regulatory complex protein	19	1.4	1	TLKDTMDSLK	-
					2	QKQEMEARER	-
Q5A0M4	*EFT2*	Elongation factor	24	4	0	DSVVAAFQWATK	-
					1	SIQRTVLMMGR	-
					2	FMDTRKDEQER	-
P30575	*ENO1*	Enolase	23	5.2	2	SERLAKLNQILR	-
					2	SGQIKTGAPARSER	-
P46598	*HSP90*	Heat shock protein 90 homolog	25	2	1	FTVTLDETNERLGR	-
P46587	*SSA2 (HSP70)*	Heat shock protein	20	4.5	1	SKVDEIVLVGGSTR	-
					0	DAVVTVPAYFNDSQR	-
Q5APD4	*MTS1*	Sphingolipid C9-methyltransferase	18	2.5	0	ITSVEMAEHVGIR	-
					0	ITSVEMAEHVGIR	-
P82612	*GPM1*	Phosphoglycerate mutase	28	4	0	NLFTGWVDVR	-
P53705	*BUD4*	Bud site selection protein	49	2.3	1	FKEVNVMSR	-
					2	KFKEVNVMSR	-
					0	SSGSSQSSLQSLR	-
					2	KESISSKPAKLSSASPR	-
Q5AIR7	*ENG1*	Endo-1,3(4)-beta-glucanase	-	-	0	TSYVQEEWK	3.00 × 10^−4^

**Table 2:** Proteins involved in adhesion were identified in the fluconazole-resistant *C. albicans* strain. Proteins identified using MASCOT are represented with sequence coverage while proteins identified using BLAST are represented with E-value.

Protein Accession	Gene Name	Protein Description	MASCOT Score	Sequence Coverage (%)	Missed Cleavage	Peptide Sequence	E-Value
Q5ABW2	*PGA60*	Putative GPI-anchored adhesin-like protein	16	2	1	MKTISILAFLVLAR	-
Q59WG7	*BIG1*	Endoplasmic reticulum protein	39	2.8	0	VEPNWNPIR	-
Q5A8T4	*ALS1*	Agglutinin-like protein	16	1.3	0	NSDAGSNGIVIVATTR	-
Q59Y31	*YWP1*	Yeast-form wall protein	-	-	0	NLYGAGAVPFFQVHLEK	2.00 × 10^−12^

**Table 3:** Proteins involved in cell wall integrity and chitin content were identified in the fluconazole-resistant *C. albicans* strain. Proteins identified using MASCOT are represented with sequence coverage while proteins identified using BLAST are represented with E-value.

Protein Accession	Gene Name	Protein Description	MASCOT Score	Sequence Coverage (%)	Missed Cleavage	Peptide Sequence	E-Value
Q5AFA2	*CRH11*	Extracellular glycosidase	55	2.4	0	SVLVADYSSGK	-
P43076	*PHR1*	pH-responsive cell surface glycosidase	-	-	1	LSYVLNQYYLDQDKK	2.00 × 10^−10^
O13318	*PHR2*	pH-responsive cell surface glycosidase	16	2.8	1	RDIPYLEAVDTNVIR	-
Q5AJC0	*UTR2*	Extracellular glycosidase	28	2.1	0	NDTWNETSNR	-
Q5A1B3	*PGA41*	Probable cell wall protein	18	2.7	0	SSYYNNLR	-
G1UB61	*CDC11*	Septin	16	2.5	1	EEQIKLEEER	-
Q5AM60	*CHT4*	Chitinase	16	2.8	2	LVELLARLRNK	-
O93852	*ALO1*	D-arabinono-1,4-lactone oxidase	27	3.8	0	FVDLTVEAGTR	-
					1	IFELNEYLKR	-
Q5AJ82	*END3*	Actin cytoskeleton-regulatory complex protein	-	-	0	DNSNNNQDNNDMLIK	4.00 × 10^−10^

**Table 4:** Efflux pumps and transporters were identified in the fluconazole-resistant *C. albicans* strain. All proteins were identified using MASCOT.

Protein Accession	Gene Name	Protein Description	MASCOT Score	Sequence Coverage (%)	Missed Cleavage	Peptide Sequence
Q5ABU7	*MDR1*	Multidrug resistance protein	34	4.6	0	ITSEGEIENSK
					2	DSFVGRVTYHLSKHK
Q5ANA3	*CDR1*	Pleiotropic ABC efflux transporter of multiple drugs	25	1	0	LVNGHALDSSFQR
Q5A762	*MLT1*	Multiple drug resistance-associated protein-like transporter	24	1	0	FYSWEKPMLAR
					1	SIKFYSWEKPMLAR

**Table 5:** Proteins involved in ergosterol biosynthesis and iron acquisition were identified in the fluconazole-resistant *C. albicans* strain. All proteins were identified using MASCOT.

Protein Accession	Gene Name	Protein Description	MASCOT Score	Sequence Coverage (%)	Missed Cleavage	Peptide Sequence
P10613	*ERG11*	Lanosterol 14-alpha demethylase	16	2.3	2	ISATYMKEIKSR
O74198	*ERG6*	Sterol 24-C-methyltransferase	24	3.7	1	SKDAASVAAEGYFK
Q5A7M3	*BNA4*	Kynurenine 3-monooxygenase	20	3.3	2	NLRSINLAVSSRGIR
G1UB63	*CSA1*	Candida surface antigen	16	2	1	SEAMKTVSSLVEVQK
Q59UP6	*PGA10 (RBT8)*	GPI-anchored protein	108	4	0	IYDQLPECAK

**Table 6:** Proteins involved in virulence were identified in the fluconazole-resistant *C. albicans* strain. All proteins were identified using MASCOT.

Protein Accession	Gene Name	Protein Description	MASCOT Score	Sequence Coverage (%)	Missed Cleavage	Peptide Sequence
P87020	*PRA1*	pH-regulated antigen	47	10.4	0	NDGWAGYWR
					2	FGSKSPFFRK
					0	TNIFWAGDLLHR
Q9P4E6	*LIP9*	Secreted lipase	21	5.1	1	FLTGDNKVFK
					1	SGWNIFKNLVVSK
Q5ANI6	*STP3*	Transcriptional regulator	17	2.5	1	SLYTISPNKGK
P43094	*SAP5*	Candidapepsin/Secreted aspartyl protease	22	2.6	0	NILYAIGAQMK
Q5AL16	*STP2*	Transcriptional regulator	16	3.8	1	ISNVKAESMK
					0	MIDPELVPFASK
P0CY31	*SEC4*	Ras-related protein	16	5.2	0	LQVWDTAGQER

**Table 7:** Proteins involved in other functions were identified in the fluconazole-resistant *C. albicans* strain. Proteins identified using MASCOT are represented with sequence coverage while proteins identified using BLAST are represented with E-value.

Protein Accession	Gene Name	Protein Description	MASCOT Score	Sequence Coverage (%)	Missed Cleavage	Peptide Sequence	E-Value
Q5A5U6	*PGA30*	GPI-anchored protein of the cell wall with unknown function	15	4.3	1	FIGGGKSSSVTK	-
Q5AEM8	*PAM17*	Presequence translocation-associated motor subunit, mitochondrial	16	5.9	1	LTWVDYFQLKK	-
P83784	*SSC1 (mtHSP70)*	Heat shock protein, mitochondrial	17	2	1	VFQGERELTR	-
Q5ALN1	*AIM36*	Altered inheritance of mitochondria protein 36, mitochondrial	16	9.6	2	EFQSYEEETGLKRR	-
					0	CLILHYDMLNELPK	-
Q5A044	*MIC60*	MICOS complex subunit	18	4.8	2	LANDWVVEGRKR	-
					0	AAISQAASNAVAMVR	-
O42766	*BMH1*	14-3-3 protein homolog	43	3.8	0	DSTLIMQLLR	-
-	*C1_13530W_A*	Pseudogene—uncharacterized	-	-	1	SGIDTTKVYGGLANYGR	3.00 × 10^−10^
Q5AB93	*PNS1*	Protein of unknown function	16	2.3	2	SAKDTFDLIRFK	-
Q59WV0	*RIM9*	pH-response regulator protein palI/RIM9	16	3.8	2	SISSRKFFESEYR	-
Q9Y7F0	*TSA1*	Peroxiredoxin	16	7.7	1	GVLRQITINDLPVGR	-
A0A1D8PDD1	*PIL1*	Echinocandin-binding protein, eisosome component	-	-	2	LSQFIKMEKNFM	2.00 × 10^−6^
Q9URB4	*FBA1*	Fructose-bisphosphate aldolase	16	3.1	0	IAEALDIFHTK	-

## Data Availability

The data presented in this study are openly available in FigShare at https://doi.org/10.6084/m9.figshare.14444651.v1 (accessed on 18 April 2021).

## References

[1] Mayer F.L., Wilson D., Hube B. (2013). *Candida albicans* pathogenicity mechanisms. Virulence.

[2] Viudes A., Pemán J., Cantón E., Ubeda P., López-Ribot J.L., Gobernado M. (2002). Candidemia at a tertiary-care hospital: Epidemiology, treatment, clinical outcome and risk factors for death. Eur. J. Clin. Microbiol. Infect. Dis..

[3] Ortega M., Marco F., Soriano A., Almela M., Martínez J.A., López J., Pitart C., Mensa J. (2011). Candida species bloodstream infection: Epidemiology and outcome in a single institution from 1991 to 2008. J. Hosp. Infect..

[4] Horn D.L., Neofytos D., Anaissie E.J., Fishman J.A., Steinbach W.J., Olyaei A.J., Marr K.A., Pfaller M.A., Chang C.H., Webster K.M. (2009). Epidemiology and outcomes of candidemia in 2019 patients: Data from the prospective antifungal therapy alliance registry. Clin. Infect. Dis..

[5] Sardi J.C.O., Scorzoni L., Bernardi T., Fusco-Almeida A.M., Mendes Giannini M.J.S. (2013). Candida species: Current epidemiology, pathogenicity, biofilm formation, natural antifungal products and new therapeutic options. J. Med. Microbiol..

[6] Bondaryk M., Kurzątkowski W., Staniszewska M. (2013). Antifungal agents commonly used in the superficial and mucosal candidiasis treatment: Mode of action and resistance development. Postepy Dermatol. Alergol..

[7] Whaley S.G., Berkow E.L., Rybak J.M., Nishimoto A.T., Barker K.S., Rogers P.D. (2017). Azole antifungal resistance in *Candida albicans* and emerging *non-albicans Candida* species. Front. Microbiol..

[8] Mansfield B.E., Oltean H.N., Oliver B.G., Hoot S.J., Leyde S.E., Hedstrom L., White T.C. (2010). Azole drugs are imported by facilitated diffusion in *Candida albicans* and other pathogenic fungi. PLoS Pathog..

[9] Hof H. (2006). A new, broad-spectrum azole antifungal: Posaconazole--mechanisms of action and resistance, spectrum of activity. Mycoses.

[10] Song J.L., Harry J.B., Eastman R.T., Oliver B.G., White T.C. (2004). The *Candida albicans* lanosterol 14-alpha-demethylase (*ERG11*) gene promoter is maximally induced after prolonged growth with antifungal drugs. Antimicrob. Agents Chemother..

[11] Sheng C., Miao Z., Ji H., Yao J., Wang W., Che X., Dong G., Lü J., Guo W., Zhang W. (2009). Three-dimensional model of lanosterol 14 alpha-demethylase from *Cryptococcus neoformans*: Active-site characterization and insights into azole binding. Antimicrob. Agents Chemother..

[12] Joseph-Horne T., Hollomon D.W. (1997). Molecular mechanisms of azole resistance in fungi. FEMS Microbiol. Lett..

[13] Berkow E.L., Lockhart S.R. (2017). Fluconazole resistance in *Candida* species: A current perspective. Infect. Drug. Resist..

[14] Morio F., Loge C., Besse B., Hennequin C., Le Pape P. (2010). Screening for amino acid substitutions in the *Candida albicans* Erg11 protein of azole-susceptible and azole-resistant clinical isolates: New substitutions and a review of the literature. Diagn. Microbiol. Infect. Dis..

[15] Prasad R., Kapoor K. (2005). Multidrug resistance in yeast *Candida*. Int. Rev. Cytol..

[16] MacPherson S., Akache B., Weber S., De Deken X., Raymond M., Turcotte B. (2005). *Candida albicans* zinc cluster protein Upc2p confers resistance to antifungal drugs and is an activator of ergosterol biosynthetic genes. Antimicrob. Agents Chemother..

[17] Hampe I.A.I., Friedman J., Edgerton M., Morschhäuser J. (2017). An acquired mechanism of antifungal drug resistance simultaneously enables *Candida albicans* to escape from intrinsic host defenses. PLoS Pathog..

[18] Casalinuovo I.A., Di Francesco P., Garaci E. (2004). Fluconazole resistance in *Candida albicans*: A review of mechanisms. Eur. Rev. Med. Pharmacol. Sci..

[19] Basma R., Barada G., Ojaimi N., Khalaf R.A. (2009). Susceptibility of *Candida albicans* to common and novel antifungal drugs, and relationship between the mating type locus and resistance, in Lebanese hospital isolates. Mycoses.

[20] Fattouh N., Hdayed D., Geukgeuzian G., Tokajian S., Khalaf R.A. Molecular mechanism of fluconazole resistance and pathogenicity attributes of Lebanese *Candida albicans* hospital isolates. Fungal Genet. Biol..

[21] Awad A., El Khoury P., Wex B., Khalaf R.A. (2018). Tandem mass spectrometric cell wall proteome profiling of a *Candida albicans hwp2* mutant strain. Curr. Mol. Pharmacol..

[22] Mrsă V., Seidl T., Gentzsch M., Tanner W. (1997). Specific labelling of cell wall proteins by biotinylation. Identification of four covalently linked O-mannosylated proteins of *Saccharomyces cerevisiae*. Yeast.

[23] Klippel N., Cui S.N., Groebe L., Bilitewski U. (2010). Deletion of the Candida albicans histidine kinase gene CHK1 improves recognition by phagocytes through an increased exposure of cell wall beta-1,3-glucans. Microbiol.-SGM.

[24] El Khoury P., Awad A., Wex B., Khalaf R.A. (2018). Proteomic analysis of a *Candida albicans pir32* null strain reveals proteins involved in adhesion, filamentation and virulence. PLoS ONE.

[25] Barrett J., Brophy P.M., Hamilton J.V. (2005). Analysing proteomic data. Int. J. Parasitol..

[26] Szklarczyk D., Gable A.L., Lyon D., Junge A., Wyder S., Huerta-Cepas J., Simonovic M., Doncheva N.T., Morris J.H., Bork P. (2019). STRING v11: Protein-protein association networks with increased coverage, supporting functional discovery in genome-wide experimental datasets. Nucleic. Acids Res..

[27] Díaz-Jiménez D.F., Pérez-García L.A., Martínez-Álvarez J.A., Mora-Montes H.M. (2012). Role of the fungal cell wall in pathogenesis and antifungal resistance. Curr. Fungal Infect. Rep..

[28] Karkowska-Kuleta J., Satala D., Bochenska O., Rapala-Kozik M., Kozik A. (2019). Moonlighting proteins are variably exposed at the cell surfaces of *Candida glabrata*, *Candida parapsilosis* and *Candida tropicalis* under certain growth conditions. BMC Microbiol..

[29] Poltermann S., Kunert A., von der Heide M., Eck R., Hartmann A., Zipfel P.F. (2007). Gpm1p is a factor H-, FHL-1-, and plasminogen-binding surface protein of *Candida albicans*. J. Biol. Chem..

[30] Copping V.M., Barelle C.J., Hube B., Gow N.A., Brown A.J., Odds F.C. (2005). Exposure of *Candida albicans* to antifungal agents affects expression of *SAP2* and *SAP9* secreted proteinase genes. J. Antimicrob. Chemother..

[31] Reyna-Beltrán E., Iranzo M., Calderón-González K.G., Mondragón-Flores R., Labra-Barrios M.L., Mormeneo S., Luna-Arias J.P. (2018). The *Candida albicans ENO1* gene encodes a transglutaminase involved in growth, cell division, morphogenesis, and osmotic protection. J. Biol. Chem..

[32] Jang H.H., Lee K.O., Chi Y.H., Jung B.G., Park S.K., Park J.H., Lee J.R., Lee S.S., Moon J.C., Yun J.W. (2004). Two enzymes in one; two yeast peroxiredoxins display oxidative stress-dependent switching from a peroxidase to a molecular chaperone function. Cell.

[33] Castillo L., Calvo E., Martínez A.I., Ruiz-Herrera J., Valentín E., Lopez J.A., Sentandreu R. (2008). A study of the *Candida albicans* cell wall proteome. Proteomics.

[34] Domínguez J.M., Martín J.J. (1998). Identification of elongation factor 2 as the essential protein targeted by sordarins in *Candida albicans*. Antimicrob. Agents Chemother..

[35] Gong Y., Li T., Yu C., Sun S. (2017). *Candida albicans* heat shock proteins and Hsps-associated signaling pathways as potential antifungal targets. Front. Cell Infect. Microbiol..

[36] Roudbarmohammadi S., Roudbary M., Bakhshi B., Katiraee F., Mohammadi R., Falahati M. (2016). *ALS1* and *ALS3* gene expression and biofilm formation in *Candida albicans* isolated from vulvovaginal candidiasis. Adv. Biomed Res..

[37] Alberti-Segui C., Morales A.J., Xing H., Kessler M.M., Willins D.A., Weinstock K.G., Cottarel G., Fechtel K., Rogers B. (2004). Identification of potential cell-surface proteins in *Candida albicans* and investigation of the role of a putative cell-surface glycosidase in adhesion and virulence. Yeast.

[38] Pardini G., De Groot P.W., Coste A.T., Karababa M., Klis F.M., de Koster C.G., Sanglard D. (2006). The CRH family coding for cell wall glycosylphosphatidylinositol proteins with a predicted transglycosidase domain affects cell wall organization and virulence of *Candida albicans*. J. Biol. Chem..

[39] Granger B.L., Flenniken M.L., Davis D.A., Mitchell A.P., Cutler J.E. (2005). Yeast wall protein 1 of *Candida albicans*. Microbiology (Reading).

[40] Granger B.L. (2012). Insight into the antiadhesive effect of yeast wall protein 1 of *Candida albicans*. Eukaryot Cell.

[41] Ha K.C., White T.C. (1999). Effects of azole antifungal drugs on the transition from yeast cells to hyphae in susceptible and resistant isolates of the pathogenic yeast *Candida albicans*. Antimicrob. Agents Chemother..

[42] Pérez A., Pedrós B., Murgui A., Casanova M., López-Ribot J.L., Martínez J.P. (2006). Biofilm formation by *Candida albicans* mutants for genes coding fungal proteins exhibiting the eight-cysteine-containing CFEM domain. FEMS Yeast Res..

[43] Sorgo A.G., Heilmann C.J., Dekker H.L., Bekker M., Brul S., de Koster C.G., de Koning L.J., Klis F.M. (2011). Effects of fluconazole on the secretome, the wall proteome, and wall integrity of the clinical fungus *Candida albicans*. Eukaryot Cell.

[44] Warenda A.J., Konopka J.B. (2002). Septin function in *Candida albicans* morphogenesis. Mol. Biol. Cell.

[45] Dünkler A., Jorde S., Wendland J. (2008). An *Ashbya gossypii cts2* mutant deficient in a sporulation-specific chitinase can be complemented by *Candida albicans CHT4*. Microbiol. Res..

[46] Martínez P., Ljungdahl P.O. (2005). Divergence of Stp1 and Stp2 transcription factors in *Candida albicans* places virulence factors required for proper nutrient acquisition under amino acid control. Mol. Cell Biol..

[47] Hoehamer C.F., Cummings E.D., Hilliard G.M., Rogers P.D. (2010). Changes in the proteome of *Candida albicans* in response to azole, polyene, and echinocandin antifungal agents. Antimicrob. Agents Chemother..

[48] Alarco A.M., Raymond M. (1999). The bZip transcription factor Cap1p is involved in multidrug resistance and oxidative stress response in *Candida albicans*. J. Bacteriol..

[49] Coste A.T., Karababa M., Ischer F., Bille J., Sanglard D. (2004). *TAC1*, transcriptional activator of *CDR* genes, is a new transcription factor involved in the regulation of *Candida albicans* ABC transporters *CDR1* and *CDR2*. Eukaryot Cell.

[50] Swoboda R.K., Bertram G., Hollander H., Greenspan D., Greenspan J.S., Gow N.A., Gooday G.W., Brown A.J. (1993). Glycolytic enzymes of *Candida albicans* are nonubiquitous immunogens during candidiasis. Infect. Immun..

[51] Newport G., Agabian N. (1997). KEX2 influences *Candida albicans* proteinase secretion and hyphal formation. J. Biol. Chem..

[52] Davis D.A., Bruno V.M., Loza L., Filler S.G., Mitchell A.P. (2002). *Candida albicans* Mds3p, a conserved regulator of pH responses and virulence identified through insertional mutagenesis. Genetics.

[53] Mille C., Bobrowicz P., Trinel P.A., Li H., Maes E., Guerardel Y., Fradin C., Martínez-Esparza M., Davidson R.C., Janbon G. (2008). Identification of a new family of genes involved in beta-1,2-mannosylation of glycans in *Pichia pastoris* and *Candida albicans*. J. Biol. Chem..

[54] Bates S., Hall R.A., Cheetham J., Netea M.G., MacCallum D.M., Brown A.J., Odds F.C., Gow N.A. (2013). Role of the *Candida albicans* MNN1 gene family in cell wall structure and virulence. BMC Res. Notes.

[55] Hall R.A., Gow N.A. (2013). Mannosylation in *Candida albicans*: Role in cell wall function and immune recognition. Mol. Microbiol..

[56] Castillo L., Martínez A.I., Garcerá A., García-Martínez J., Ruiz-Herrera J., Valentín E., Sentandreu R. (2006). Genomic response programs of *Candida albicans* following protoplasting and regeneration. Fungal Genet. Biol..

[57] Zohbi R., Wex B., Khalaf R.A. (2014). Comparative proteomic analysis of a *Candida albicans DSE1* mutant under filamentous and non-filamentous conditions. Yeast.

[58] El Khoury P., Salameh C., Younes S., Awad A., Said Y., Khalaf R.A. (2019). Phenotypic and Cell Wall Proteomic Characterization of a *DDR48* Mutant *Candida albicans* Strain. J. Microbiol. Biotechnol..

[59] Awad A., El Khoury P., Wex B., Khalaf R.A. (2018). Proteomic analysis of a *Candida albicans* pga1 Null Strain. EuPA Open Proteom..

